# A single-center, retrospective, cross-sectional study comparing the number of non-diagnostic measurements ratio in the pSWE and SSI ultrasound elastography methods

**DOI:** 10.1097/MD.0000000000033964

**Published:** 2023-06-02

**Authors:** Maciej Cebula, Jakub Kufel, Katarzyna Gruszczyńska

**Affiliations:** a Department of Radiodiagnostics, Invasive Radiology and Nuclear Medicine, Department of Radiology and Nuclear Medicine, Faculty of Medical Sciences in Katowice, Medical University of Silesia, Katowice, Poland; b Department of Biophysics, Faculty of Medical Sciences in Zabrze, Medical University of Silesia, Zabrze, Poland; c Department of Diagnostic Imaging, Department of Radiology and Nuclear Medicine, Faculty of Medical Sciences in Katowice, Medical University of Silesia, Katowice, Poland.

**Keywords:** liver, point shear wave elastography, supersonic shear imaging, ultrasound elastography

## Abstract

The point shear wave elastography and supersonic shear imaging methods were compared regarding incorrect measurements during the liver examinations. A report-based, single-center, retrospective analysis of 425 liver elastography examinations was performed. A lower success ratio was observed for the point shear wave elastography method, as well as the older and obese patients pre-dominated in non-diagnostic studies. In our center experience, it is easier to obtain diagnostic data using the supersonic shear imaging method. However, further investigation of the subject is needed.

## 1. Introduction

Ultrasound elastography was first described in the 1990s and constantly growing family of diagnostic methods.^[1]^ In recent years have been constant development and improvement to enable the quantification of tissue stiffness. Elastography methods use the variable elasticity of tissues resulting from specific diseases and physiological processes.^[2]^ It is well known that many solid tumors differ mechanically and therefore also in flexibility from the surrounding healthy soft tissues. Likewise, with liver disease processes, in which the pathological tissue becomes less compliant and less flexible than the surrounding non-affected soft tissues.^[3]^ Currently, the clinical applications of elastographic methods is much wider, allowing for the diagnosis of numerous areas of the body, such as muscles, breast, thyroid, spleen, pancreas or intestines.^[4–11]^

Accordingly, elastographic methods can be used to distinguish between tissues affected by a disease process resulting in reduced tissue elasticity from healthy tissues.

Elastography found a place in the diagnostic and control of liver diseases, reducing the need for this organ biopsy.^[12,13]^ The first introduced method was a FibroScan (Echosens, Paris, France), which efficiency and reproducibility was confirmed by numerous studies.^[14–17]^ It was followed by Acoustic Radiation Force Impulse offered with Acuson S2000 ultrasound device (Siemens Medical Solutions, Mountain view, CA). The method introduced selection of the measurement site controlled by real-time B-mode image. Next step was a point shear wave elastography (pSWE), that offers local shear wave speed measurement with a reliability indicator for each measurement.^[18]^ The method extension, a 2-D Shear Wave elastography and Supersonic Shear Imaging (SSI) were next introduced, offering static or real-time evaluation of shear wave speed of whole tissue areas.^[19]^ It is worth mentioning that various other strategies for quantifying liver function are currently being explored^[20–23]^ as well as its cost-benefit analysis.^[24]^

**Figure 1.**
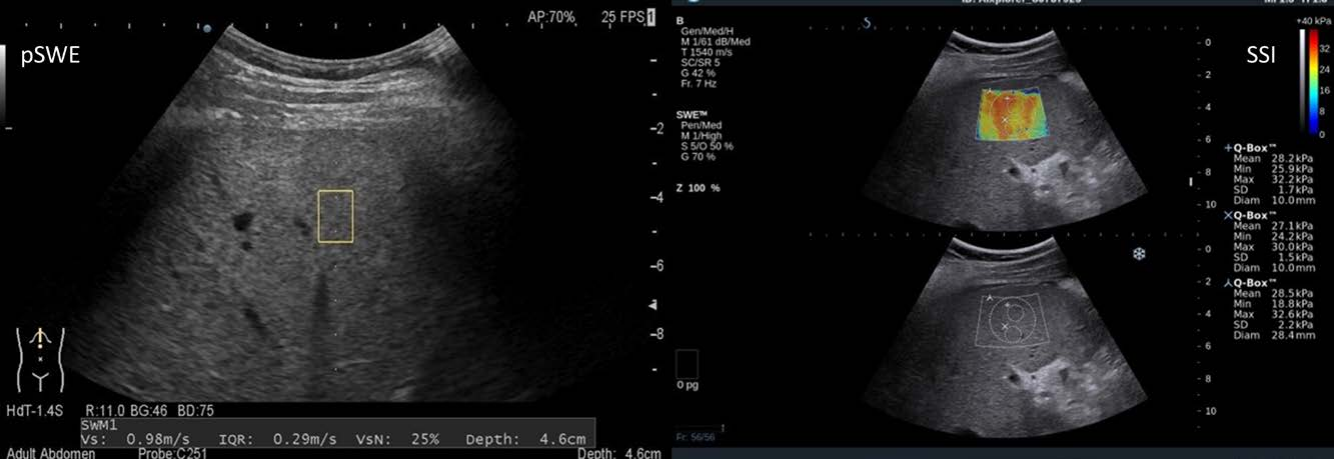
The image obtained during the examination using the pSWE (left) and SSI (right) methods together with the quantitative variables offered by these methods. pSWE = point shear wave elastography, SSI = supersonic shear imaging.

Many ultrasound vendors introduce their varieties, so the need for standardization and comparison is on the rise. As most of the researchers focus on accuracy, the authors of this paper decided to compare the pSWE with SSI methods regarding ease of use and ratio of incorrect measurements during the liver examinations, as both methods are available in our setting. The above techniques differ in how they are performed and how physicians perceive their difficulty.^[25]^ The success ratios of forementioned methods differ and are affected by numerous factors,^[26]^ so it was decided to undertake a retrospective analysis to look closer at the issue. The image differences and the range of data offered between the pSWE and SSI methods are visualized in Figure 1.

The main purpose of the work is to compare the percentage of diagnostic and non-diagnostic measurements in the SSI and pSWE methods, and the secondary goal is to search for factors that may be responsible for the non-diagnosticity of measurements based on the available data.

## 2. Materials and methods

The anonymized data of all performed in the Department of Radiodiagnostics and Invasive Radiology of Central Clinical Hospital of Prof K. Gibiński of the Medical University of Silesia in Katowice, Poland liver elastographies was gathered. The analysis covered March 2018 to August 2021 period. Based on anonymized examination reports, the information about the date, diagnosis, patients weight and height, measurement method and device, performing physician, quality assurance parameters, i.e., reliability indicator and interquartile range to median ratio (IQR/M) for pSWE and stability index (SI) for SSI. The results of 425 examinations, 317 pSWE and 108 SSI exams were gathered. The body mass index (BMI) has been calculated, and patients with BMI ≥ 30 kg/m^2^ were classified as obese.

The study included in- and out-hospital adult patients of both genders and all underlying diffused liver diseases. A lack or insufficient detail of information in the report regarding collected data was the only exclusion criterium, and none of the reports was rejected.

The pSWE examinations were performed on Hitachi Aloka Arietta V70 ultra-sound with C251 (1.8–5MHz) convex probe in accordance to European Federation of Societies for Ultrasound in Medicine and Biology Guidelines and Recommendations (Update, 2017)^[19]^ and Shear Wave Measurement Section Ver. 3.0 Instruction Manual.^[26]^ The cutoffs of 60% for reliability indicator and IQR/M ≤ 1/3 were used.

The SSI examinations were performed on Hologic Supersonic Mach 30 ultrasound with C6-1X convex probe in accordance to European Federation of Societies for Ultra-sound in Medicine and Biology Guidelines and Recommendations (Update, 2017)^[19]^ and Aixplorer MACH Protocols.^[27]^ The cutoff of 90% for SI was used.

Examinations that did not meet the cutoffs were classified as non-diagnostic. All the studies were performed by the group of physicians composed of 8 radiology specialists and 9 radiology residents who underwent theoretical and practical training for each method.

The statistical analysis was performed with Statistica 13.0 software.^[28]^ The quantitative variables were presented as an arithmetic mean and a standard deviation (normally distributed variable) or a median and the interquartile range (variables of not normal/skewed distribution). The normality of distribution was assessed with the Shapiro–Wilk test. Qualitative variables were presented as absolute values and percentages. The intergroup differences for the quantitative variable were evaluated with the Mann–Whitney *U* test. Fisher’s exact test was performed for qualitative variables. Statistical significance was established at *P* < .05.

Due to the retrospective character and patient data anonymization, ethical review and approval were waived for this study by Ethics Committee of Medical University of Silesia.

## 3. Results

The study group consisted of 425 patients; 241 females (56.7%) and 184 males (43.3%), aged 54.94 ± 14.79 years (56.68 ± 14.35 years and 52.66 ± 15.01 years, respectively). The characteristics of the study group are shown in Table 1.

**Table 1:** Demographic of the study group.

Variable	pSWE (n = 317)	SSI (n = 108)	*P* value
Gender [n (%)]			
Females	175 (55.20)	66 (61.11)	.169
Males	142 (44.80)	42 (38.89)	
Age [yr]	54.17 ± 14.81	57.19 ± 14.60	.085
Weight [kg]	76.09 ± 13.81	75.64 ± 9.22	.905
Height [cm]	167.38 ± 8.50	167.51 ± 4.44	.667
BMI [kg/m^2^]	27.12 ± 4.21	26.95 ± 2.95	.808
Obese [n (%)]	76 (23.97)	14 (12.96)	.016
Diagnostics [n (%)]			
Diagnostic	226 (71.29)	103 (95.37)	<.001
Non-diagnostic	91 (28.71)	5 (4.63)	

BMI = body mass index, pSWE = point shear wave elastography, SSI = supersonic shear imaging.

**Figure 2.**
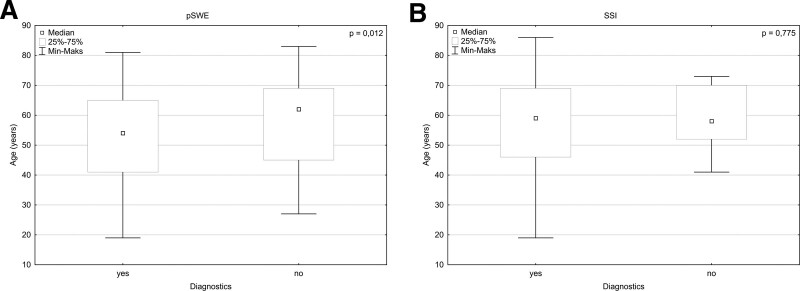
Age difference in non- and diagnostic groups divided by elastography method: (A) Age difference between the non- and diagnostic group in point shear wave elastography method; (B) Age difference between the non- and diagnostic group in supersonic shear imaging method.

A significant difference in age between genders has been observed (*P* = .003), with females being older. The age difference between non- and diagnostic groups was significant (*P* = .047), but it was observed only in pSWE (*P* = .012), not SSI examinations (*P* = .775) after division into method-based subgroups. The older patients predominated in the group of non-diagnostic studies; this relation is presented in Figure 2.

**Figure 3.**
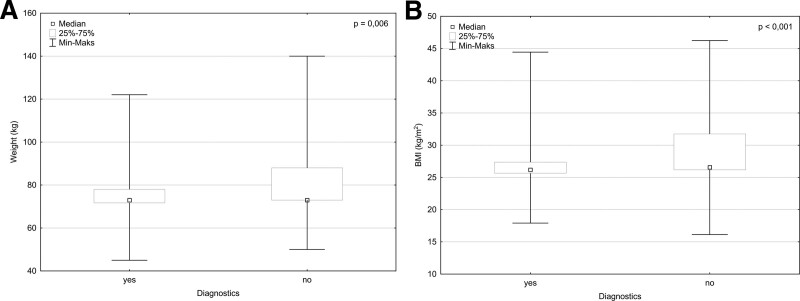
Age difference in non- and diagnostic groups: (A) Weight difference between the non- and diagnostic group in both elastography methods; (B) BMI difference between the non- and diagnostic group in both elastography methods. BMI = body mass index.

There was no significant difference in genders distribution among non- and diagnostic groups (*P* = .505). The patients did not differ in weight (*P* = .905), height (*P* = .667) and BMI (*P* = .808) between the elastography methods, while a significant difference was observed in weight (*P* = .006) and BMI (*P* < .001) between non- and diagnostic groups; results are presented in Figure 3.

Additionally, when grouped for the elastography method, above differences for weight and BMI were still present for the pSWE with *P* = .011 and *P* = .001 respectively, while absent for the SSI with *P* = .184 and *P* = .133 respectively. Further analysis showed significantly higher amount of obese patients in non-diagnostic (*P* < .001) and pSWE (*P* = .016) subgroups.

## 4. Discussion

The current scientific reports claim that reliable measurements can be obtained in 90% to 95% of patients with pSWE^[29–32]^ and around 90% with the SSI method.^[25,33–36]^ In our case, these percentages were 71.29% and 95.37%, respectively. The lower success ratio in pSWE could result from a significantly older group of patients. Still, up-to-date literature is inconsistent with the influence of age on the reproducibility of these methods.^[37,38]^ It seems that more research is needed on this topic, with a structured methodology allowing for more consistent results from the presented methods.

The gender does not seem to affect the success ratio of either analyzed method, which is consistent with current reports.^[39,40]^

In analyzed group the mean BMI was 27.07 kg/m^2^ and included 90 patients (21.17%) with BMI ≥ 30 kg/m^2^. As the weight, height, and BMI did not differ significantly between pSWE and SSI subgroups, the reason for discrepancy in amount of non-diagnostic measurements can be due to obese patients distribution. Our group is comparable to one presented by Cassinotto et al^[25]^ in regard of the mean BMI (27.4 k/m^2^) and they reported failure ratio of 10.4% for the SSI method, which is higher than observed in our study. This can be due to higher percentage of obese patients (30.37%) compared to our group (21.18%). The authors reported higher ratio of failures in the obese patients, and We were able to confirm this observation. The results presented by Leung et al^[41]^ as well Ferraioli et al^[42]^ presented lower failure ratios in SSI, 1.1% and 2.5% respectively, as both studies included groups with lower BMI (appropriately 24.2 kg/m^2^ and 25.4 kg/m^2^).

The Giuffrè et al^[43]^ presented results of the bariatric patients study based on pSWE method, where mean BMI was 44 kg/m^2^ and failure ratio of 7%. As it is reported, that the pSWE method seems to be less influenced by obesity,^[44]^ the results of our study seems to be contraindicatory. The surprisingly high ratio of failed pSWE could be due to different composition of underlying pathologies or ultrasound device choice, but reliable analysis seems impossible due to nature of our study.

Unfortunately, the aspect that is under-investigated in this study is a learning curve for each of the methods and operators. The existing reports claim that a 1-year training or 130 examinations are needed for the pSWE^[45]^ and 50 studies for the SSI method.^[46]^ In our case, all of the operators finished theoretical and practical training for each technique. Most of them use elastography outside of our center, obtaining a number of procedures performed that exceeds the listed values. Additionally, the non-diagnostic cases are somewhere about evenly time distributed, which contradicts the operator fault.

The main limitations of this study are single-center character, lack of additional, relevant data regarding measurement quality, and comparison of only one system capable of pSWE measurements with SSI. A prospective, multi-center study would provide a more accurate answer to the analyzed problem. Still, due to the inability of direct results comparison between ultrasound systems,^[19,47]^ the need for identically equipped departments inclusion can be a severe obstacle to realizing such a project. My work also lacks data on the needed amount of pSWE or SSI measurements performed to fulfill the guidelines’ requirements. More failed and suboptimal measurement attempts resulting in longer examination time seem like an essential variable in future problem studies. The comparison analysis of pSWE capable ultrasound systems has focused on the comparability of measurements and accuracy,^[48–51]^ not the error or success ratios. This aspect seems to be worth of further investigation.

## 5. Conclusions

The experience of our center shows that the percentage of diagnostic measurements is higher in the SSI method in relation to the pSWE. However, a prospective, multicentre study comparing more commercially available ultrasound machines offering elastography would be necessary to assess the repeatability and reproducibility of measurements. The optimal methodology seems to be to compare the same group of patients on different devices, which unfortunately was not possible in our study.

## Author contributions

**Conceptualization:** Maciej Cebula.

**Data curation:** Maciej Cebula.

**Formal analysis:** Maciej Cebula.

**Funding acquisition:** Maciej Cebula.

**Investigation:** Maciej Cebula.

**Methodology:** Maciej Cebula.

**Project administration:** Maciej Cebula.

**Resources:** Katarzyna Gruszczyńska.

**Supervision:** Katarzyna Gruszczyńska.

**Validation:** Maciej Cebula, Katarzyna Gruszczyńska.

**Visualization:** Maciej Cebula.

**Writing – original draft:** Maciej Cebula.

**Writing – review & editing:** Maciej Cebula, Jakub Kufel.
